# Properties of sandcrete blocks stabilized with cashew apple ash as a partial replacement for cement

**DOI:** 10.1038/s41598-024-55031-7

**Published:** 2024-03-21

**Authors:** Peter Korankye, Humphrey Danso

**Affiliations:** 1 Department of Construction Technology and Management Education, Akenten Appiah-Menka University of Skills Training and Entrepreneurial Development, P. O. Box 1277, Kumasi, Ghana; 2 Department of Architecture and Civil Engineering, Akenten Appiah-Menka University of Skills Training and Entrepreneurial Development, P. O. Box 1277, Kumasi, Ghana

**Keywords:** Cashew apple ash, Cement, Compressive strength, Sandcrete block, Split tensile strength, Civil engineering, Mechanical properties

## Abstract

The use of by-products from agricultural production as stabilizers in concrete and mortar in developing countries could result in numerous benefits. These by-products are readily available, cheap, and have a lesser carbon footprint. As Portland cement prices keep rising, the search for alternatives to sustainable construction materials is necessary. Cashew apples are left on cashew farms as waste material after the nuts have been removed due to lack of utilization. In this study, the properties of sandcrete blocks produced with cashew apple ash (CAA) as a partial replacement for cement were investigated. A total of 180 block specimens of size 100 × 100 × 130 mm were prepared from six different mortar mixes of control, 5, 10, 15, 20, and 25% CAA replacement of cement by weight were prepared. Results revealed that the highest compressive and tensile strengths after 28 days of curing CAA blocks were 11.45 and 1.08 N/mm^2^ respectively. The best water absorption resistance obtained was 2.66%. The study recommends the use of 5% CAA replacement of cement to block manufacturers for use in manufacturing sandcrete blocks. This study is useful because the cashew apple waste ash used as an alternative material to cement in sandcrete block production will be beneficial to the environment and may also save the cost of production of sandcrete blocks.

## Introduction

Housing can be described as an essential component, acknowledged universally as the second most important need of humans after food^1,2^. Ghana's construction sector contributes significantly to gross domestic product and employment. Cement is one of the major used construction materials in Ghana. It serves as a binding agent for building materials like concrete, sandcrete bricks and blocks, and cement mortar. Due to the high cost of cement, researchers are working on alternatives to reduce the quantity of cement used in sandcrete block production^3,4^. Sandcrete blocks and bricks are commonly used as walling units in Ghana, Nigeria, and other African nations, with over 90% of physical infrastructure constructed using sandcrete blocks produced manually or mechanically^5^. As a result, sandcrete blocks are considered a vital component of Ghana's construction industry.

Sandcrete blocks are durable but costly, leading to the search for low-cost alternatives. They are essential for socio-economic development and resistant to degrading, rusting, and insect attack. Olubajo et al*.*^6^ established that the availability of an alternative to these construction materials is highly essential as a stimulant for socio-economic development. Researchers are exploring supplementary cement replacement materials such as agricultural and mineral pozzolanas as partial cement replacement materials in sandcrete block production to reduce binder costs and address agro-waste disposal challenges^7^.

Studies^6–8^ have suggested that calcined agro-waste can be used as a partial replacement for cement with pozzolana, reducing the cost of sandcrete blocks and cementitious composites. Pozzolana, as defined by Olubajo et al.^6^ is a siliceous substance, which reacts with lime to create pozzolanic compounds. According to Anowai et al.^7^, maize cob ash can partially replace cement in sandcrete blocks with about 10 to 20% quantities. Manasseh^8^ discovered that agro-based by-products such as rice husk ash, regarded as waste in many underdeveloped societies, can be used as a partial replacement for Portland cement for the manufacturing of sandcrete blocks.Apata and Alhassan^9^ found that 10% of locally available waste material can replace ordinary Portland cement (OPC) for low-cost housing.

Previous studies^10–12^ have shown promising results in using pozzolanic ashes from agricultural by-products as a partial replacement for cement in the construction industry for producing sandcrete blocks. Olawale and Tijani^10^ found that adding 3% cocoa pod husk ash (CPHA) to the local procedure for producing sandcrete blocks increased the compressive strength by over 100%. Arasa and Onchiri^11^ found that up to 15% of sugarcane bagasse ash (SBA) used as a replacement for cement for sandcrete block production satisfied the 3.45 N/mm^2^ compressive strength recommended standards for load-bearing walls. Anowai and Afunanya^12^ also found that the compressive strength of sandcrete blocks with 10 and 20% millet husk ash replacement of cement and water absorption were within acceptable limits. This implies that other agro-waste products can be investigated as cement replacement materials for producing sandcrete blocks for low-cost housing units. The mechanism of action of agricultural waste to replace some cementitious materials is an ongoing debate in materials science.

Ghana is among the West African nations that produce the most cashews. In 2019, Ghana contributed 171,924 metric tons of cashew nuts to the global market^13^. Cashew is one of the non-traditional export crops that could change Ghana's economy if its popularity increases rapidly and governments support its production^14^. According to Statista^15^ Ghana exported 302,000 metric tons of cashew nuts in 2020, of which the largest proportion was unshelled. One of the major problems is that greater portions of cashew apples are wasted because they cannot be used, unlike cashew nut kernels, which directly decrease farmers' incomes. Decomposition in the plantation emits an unpleasant odour that harms the environment. Hence, value addition or proper disposal of cashew apple waste is essential to ascertain its suitability in the construction industry^16^. This would be an effective and economical solution for disposing of this agro-waste, and it could also reduce CO_2_ emissions associated with cement production as well as the cost of cementitious composites in the construction industry. Additionally, the use of calcined cashew apple waste as a partial replacement for cement can also be used as an alternative substitute for cement in sandcrete block production, as this experimental study explored.

A number of research works^17–19^ have been done on the effects of cashew nutshell ash on concrete production, contributing not only to the valorization of a cashew but also to the reduction of the overall carbon footprint of cementitious systems. A study^17^ investigated the flexural strength of concrete stabilized with cashew nutshell through modelling and found that the strength can be predicted so as to save both money and time when conducting experimental studies. Another study^18^ determined the strength and workability of concrete incorporating anacardium occidentale nutshell ash and found a 5% increase in compressive strength and a high precision between the optimisation slump and the experimental slump with a strong relationship between the model equations. Oyebisi et al*.*^19^ examined the properties of concrete produced with cashew nutshell waste ash as replacement of limestone cement at 5, 10, 15 and 20%, and recommended that 15 and 20% replacement for structural application and non-load bearing application respectively. However, little or no study has been conducted on using cashew apple ash as a substitution of cement in producing sandcrete blocks. This study seeks to address this gap in literature.

Therefore, a study on replacing cement with solid agro-waste ash from the calcination of cashew apples on the properties of sandcrete blocks can be investigated as a construction material. By using this material, the quantity of cement production and use will reduced, thereby promoting sustainability, and reducing environmental problems. The rising cost of cement for producing sandcrete blocks has adversely affected the construction industry in developing countries. Therefore, a study on alternative materials such as cashew apple ash in the construction industry justified this study. This study aims to investigate the properties of sandcrete blocks produced with cashew apple ash (CAA) as a replacement for cement. In this present study, an experimental research design was used to determine the properties of sandcrete blocks manufactured with sand, cement and partial replacement of cement with CAA, and water. The properties of the sandcrete blocks determined were density, water absorption, compressive strength, and split tensile strength. Six different mortar mixes of 0 (control), 5, 10, 15, 20, and 25% CAA replacement of cement by weight were used.

## Materials and methods

### Materials

Cement, sand, cashew apple, and water were the raw materials used for this study which investigated the properties of sandcrete blocks stabilized with cashew apple ash as a partial replacement for cement.

#### Cement

The cement utilized for the experiment was ordinary Portland cement produced by Ghana Cement Limited (GHACEM), Ghana’s largest cement producer and the most common type of cement readily available in almost every part of Ghana. The cement conforms to the British Standard BS 12:1996^20^ and meets the requirements of Ghana Standard GS 766:2011^21^, classified as grade 32.5R.

#### Fine aggregate (sand)

Natural pit sand obtained from a construction site at Achinakrom located in Ejisu Municipality in the Ashanti Region of Ghana was used for the experimental work. The sand was free of clay, silt, dirt, and organic matter. It is commonly used as fine aggregate for sandcrete block production in Kumasi in the Ashanti Region of Ghana.

#### Water

Portable drinking water from the tap was used to prepare the mixes for the experiment since it does not contain any soluble iron or metals that may affect the sandcrete block's ability to set and hydrate by BS 3148 Part 2: 1980^22^. The water used was obtained from the Construction Departmental Laboratory at the Akenten Appiah-Minka University of Skills and Entrepreneurial Development, Kumasi, where the experimental study was conducted.

#### Cashew apples

**Figure 1:**
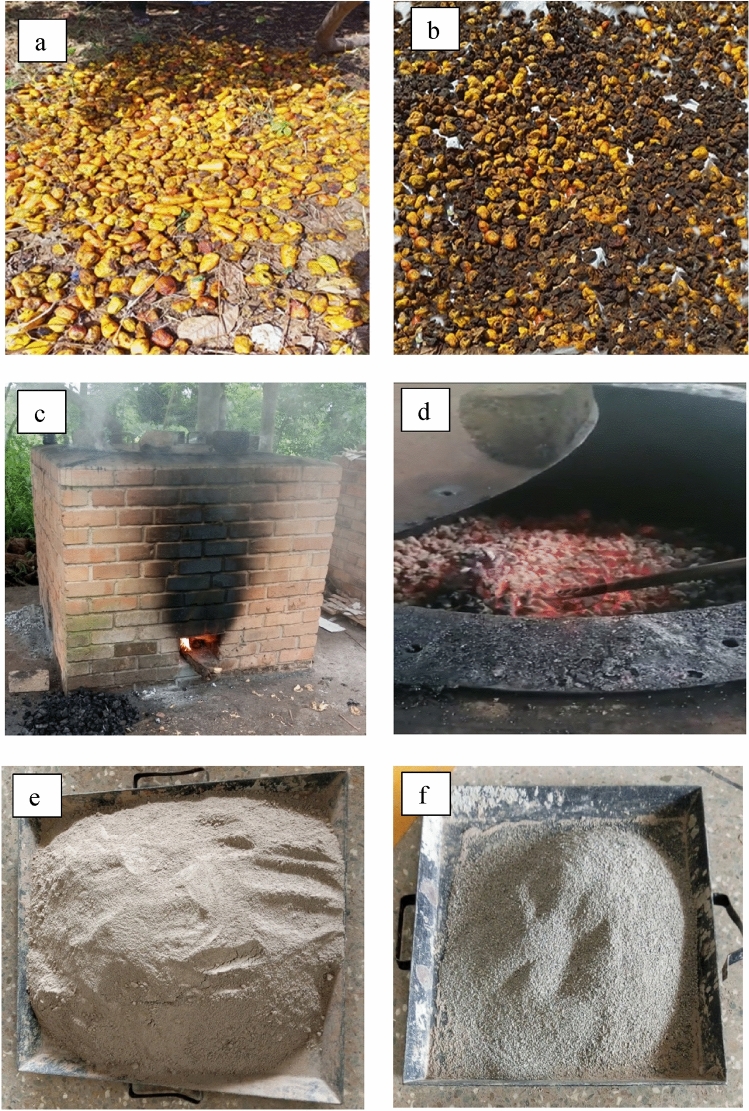
Production process of cashew apple ash: (**a**) cashew apple waste, (**b**) sun-drying of cashew apple waste, (**c**) burning of cashew apple waste in a locally built furnace, (**d**) burning of cashew apple waste at temperature of 735 °C, (**e**) cashew apple ash ground into fine particles, (**f**) sieved cashew apple ash.

The waste cashew apples used for this study were locally sourced from farmers in Tanoboase of Techiman North Municipal in the Bono East Region of Ghana. Cashew is the primary cash crop that supports the majority of households in the area. The region is known for its suitable climatic conditions and fertile soils that support the growth and production of cashews. The cashew apple waste used in the study was collected directly from the cashew farms of the local farmers. The cashew apple waste was harvested daily by picking them from the ground after farmers separated the nuts and left them on the ground to go to waste. This is shown in Fig. 1a.

### Material preparation and testing of specimen

#### Preparation of cashew apple ash (CAA)

Apple juice was squeezed out to reduce moisture content for speedy drying of the cashew apples’ waste (Fig. 1a). The cashew apples were dried under the sun (Fig. 1b) for 42 days, approximately one and a half months for easy burning. The cashew apples were burned through a locally built furnace (Fig. 1c) to obtain the ash. The burning took almost one and a half days to produce the ash. The initial temperature was 400 °C was increased to reach the desired temperature of 735 °C. The heat was maintained at this temperature for 32 h (Fig. 1d). The oven was put off and kept closed for another 24 h for cooling. After cooling the ash samples were collected, ground into fine particles (Fig. 1e), and then sieved using a 600-micron sieve to remove thicker and impure particles. The fine particles (Fig. 1f) were used for the experimental work. The particle size of the CAA used was influenced by a prvious study^23^.

#### Chemical composition of CAA and OPC

The chemical composition of CAA and OPC was determined using an X-ray fluorescence technique to identify the elemental chemical constituents of CAA and Ghacem OPC 32.5R. The CAA used in this study satisfied the chemical composition criterion as the sum of silica (SiO_2_), alumina (Al_2_O_3_), and iron oxide (Fe_2_O_3_) contents were more than the specified minimum of 70% as per ASTM C618 –19^24^.

#### Mortar mix design and proportion

A mix ratio of 1:6 and a water-cement ratio of 0.5. The cement content was replaced by CAA at 0 to 25% at 5% intervals. These mix designs were used for the experimental work with their quantities as shown in Table 1.

**Table 1:** Mix design and quantity of materials.

Mix design (%)	Quantity of materials (kg)			
	OPC	CAA	Sand	Water
0	11.13	0	66.703	5.565
5	10.56	0.57	66.703	5.565
10	10.02	1.11	66.703	5.565
15	9.45	1.68	66.703	5.565
20	8.91	2.22	66.703	5.565
25	8.34	2.79	66.703	5.565

### Specimens preparation

**Figure 2:**
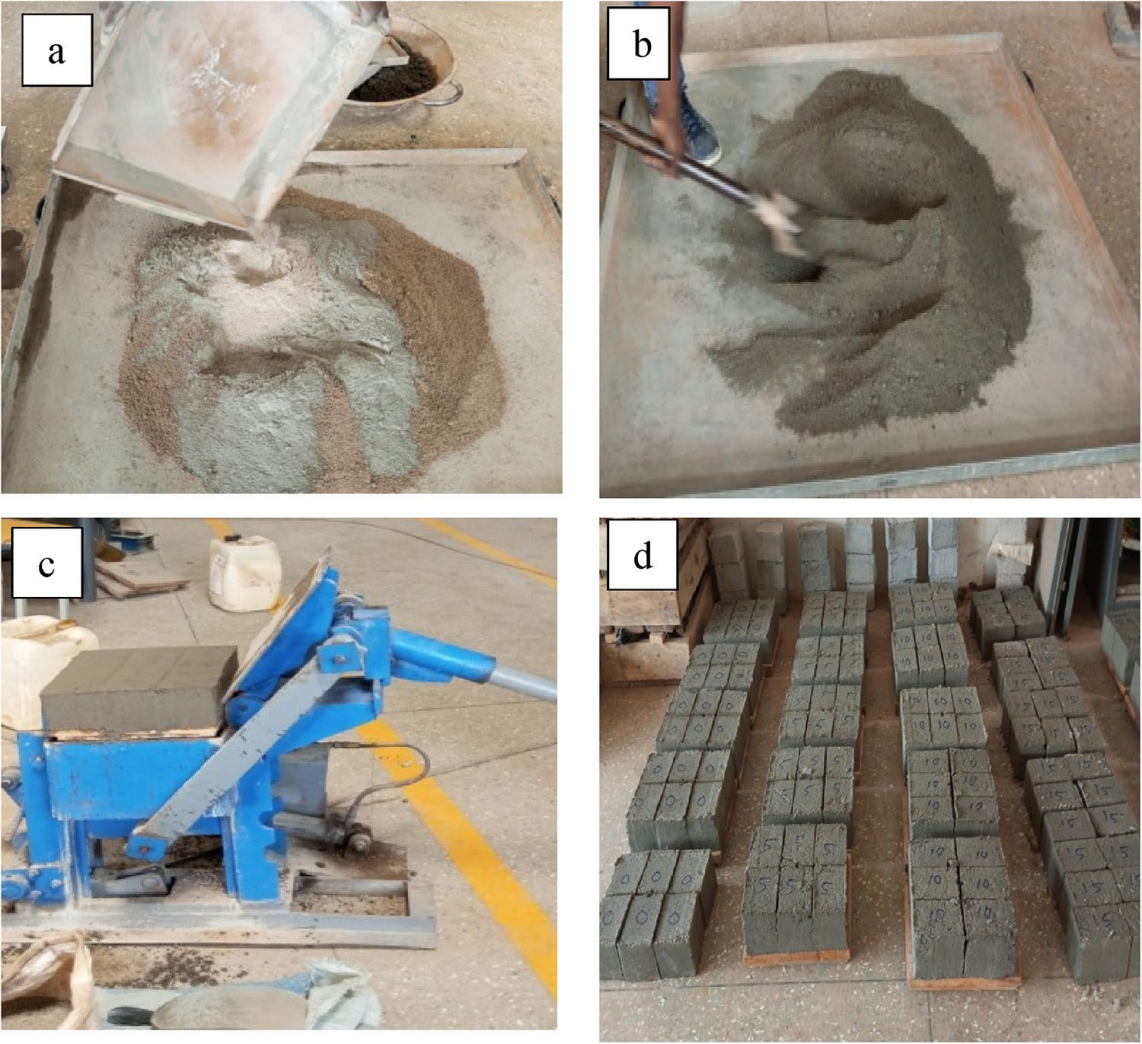
Production process of sancrete block specimens: (**a**) spreading of cement and CAA on the sand, (**b**) mixing of the materials, (**c**) moulding of the block specimens using compressed block making machine, (**d**) curing of the block specimens.

The study employed a water-cement ratio of 0.5 by weight and a mix ratio of 1:6 in producing the sandcrete block specimens. CAA was used to replace ordinary Portland cement in sandcrete blocks at five different levels: 5, 10, 15, 20, and 25%. The control specimens had 0% replacement of cement with CAA. The calculated quantity of materials for each mix design (as shown in Table 1) was batched by weight. The cement and CAA were spread on the sand (Fig. 2a) and manually mixed with a spade on a metal platform to obtain a homogenous mixture (Fig. 2b). Water was sprinkled on the mixture to obtain the mortar. A compressed block moulding machine was used to mould the block specimens of size 130 × 100 × 100 mm (Fig. 2c). In all, 180 block specimens were produced and cured for 7, 14, 21, and 28 days using sprinkling method (Fig. 2d). The specimens were tested for density, compressive strength, split tensile strength, and water absorption at the curing ages.

### Testing of block specimens

#### Density

The density of the block specimens was determined following BS 2028:2000^25^. The mass of the block specimens was measured with a weight balance after the specimens were oven-dried. The volume of the block specimens was determined. The densities of the block specimens were calculated with the Eq. (1).

1 $$\uprho =\frac{{\text{m}}}{\mathrm{V }},$$

where: $\uprho$ = density (kg/m^3^); m = mass of block specimen (kg); V = volume of block specimen (m^3^).

#### Water absorption test

A water absorption test was performed following BS 1881–122:2011^26^ to determine the rate of intake of water by the sandcrete block specimens produced with partial replacement of CAA. In conducting the test, the mass of each block specimen was measured after oven-drying, and immersed in water for 10 min, after which the mass of water absorbed was specimen also measured. Water absorption was then calculated with the Eq. (2).

2 $$WA =\frac{WA}{ M }\times 100\mathrm{\% },$$

where: WA = water absorption (%); M = mass of the oven-dried block specimen (kg); WA = mass of water absorbed block specimen (kg).

#### Compressive strength test

**Figure 3:**
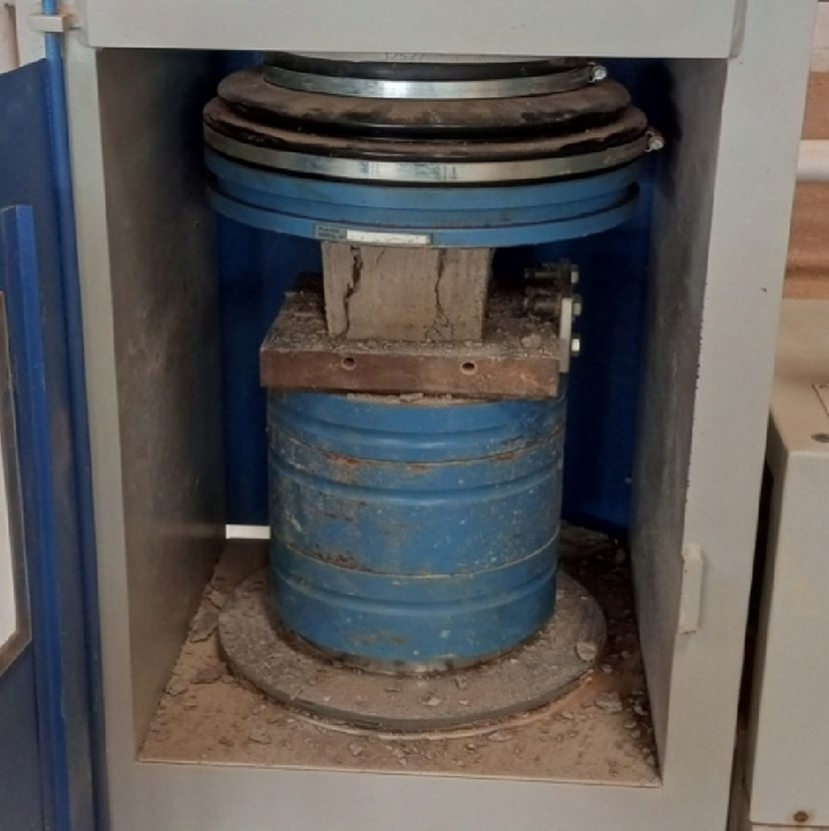
Specimen failed under compressive strength test.

A compressive strength test was conducted to determine the load-carrying strength of the block specimens. This was done following BS EN 772–1:2011^27^. The block specimens were cleaned with a duster after curing. Each block specimen was placed in the compression testing machine and load was applied until it failed (Fig. 3). The compressive strength was calculated by the Eq. (3).

3 $${f}_{c} = \frac{F}{A} ,$$

where: f_c_ = compressive strength (N/mm^2^); F = maximum force at which the specimen failed (N); A = cross-sectional area of the specimen (mm^2^).

#### Splitting tensile strength test

**Figure 4:**
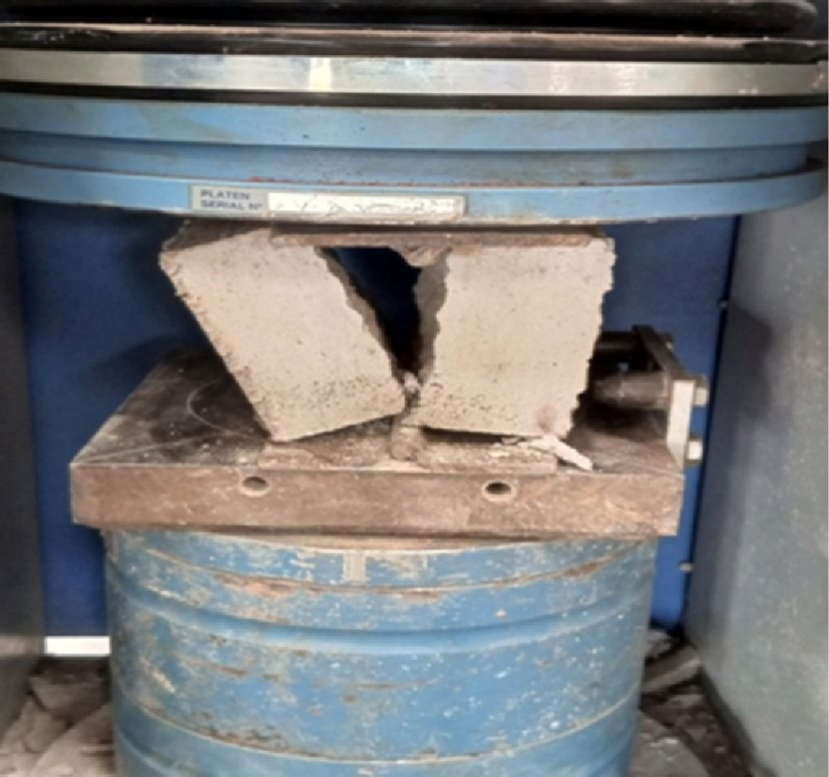
Split block specimen under tensile strength test.

Splitting tensile strength test was performed following BS EN 12390-6:2009^28^. The block specimens were placed in the compression testing machine with a splitting jig placed centrally below and above the block specimen. The load was applied on the block specimen until it split into two (Fig. 4). The splitting tensile strength of the blocks was determined with the Eq. (4).

4 $${f}_{t}= \frac{2P}{\pi Ld} ,$$

where: f_t_ = splitting tensile strength (N/mm^2^); P = maximum force at which the specimen split (N); L = length of the specimen (mm); d = width of the specimen (mm).

### Statement of plant material used

The authors confirm that plant material (cashew apple) used in the study comply with the relevant institutional and national (Ghana) guidelines and legislation.

## Results and discussion

### Chemical composition of CAA and OPC

Table 2 presents the results of the chemical analysis of Cashew Apple Ash and Ordinary Portland Cement. The results revealed that the sum of Silicon Oxide (SiO_2_), Aluminium Oxide (Al_2_O_3_), and Iron Oxide (Fe_2_O_3_) is 80.5% for CAA, which is far above the 70% minimum specified by ASTM C618-19^24^ for pozzolanas. The loss on ignition for CAA is 11.5%, which is less than the maximum value of 12% specified by ASTM C311-07^29^. The OPC had the sum of Silicon Oxide (SiO_2_), Aluminium Oxide (Al_2_O_3_), and Iron Oxide (Fe_2_O_3_) to be 27.09% and with Calcium oxide (CaO) of 57.82%. The result showed that CAA, which is viewed as waste on cashew farms, is pozzolanic and can be used as a partial replacement for cement in the production of sandcrete blocks. The combined chemical composition result of SiO_2_ + Al_2_O_3_ + Fe_2_O_3_ of the CAA was higher than the OPC (27.09%) and 27.70% obtained by Olubajo et al.^6^ study with orange peel ash (OPA) as a partial cement replacement, 28.9% found in the study conducted by Afolayan et al.^30^. The combined chemical composition result of the CAA (80.5%) concurs with Elinwa and Ejeh’s^31^ study had 73.07% from sawdust ash (SDA) which was higher than the 70% minimum required by ASTM C618-19^24^.

**Table 2:** Chemical composition of CAA and OPC.

Parameters	Composition (%)	
	CAA	OPC
SiO_2_	38.36	18.66
Al_2_O_3_	42.14	5.58
Fe_2_O_3_	0.01	2.85
CaO	6.34	57.82
MgO	4.26	7.46
Na_2_O	0.33	0.15
K_2_O	4.10	1.15
SO_3_	0.12	2.56
LOI	11.46	8.79

### Density of sandcrete block stabilized with cashew apple ash

Table 3 presents the average density of sandcrete block stabilized with CAA as a partial replacement for cement. The results range from 2134.615 to 2288.462 kg/m^3^, which is similar to the result obtained by Raheem et al.^32^, with a density ranging from 2146.46 kg/m^3^ to 2209.60 kg/m^3^. These values are slightly lower than the densities found in the study by Kumari et al.^33^, which ranged from 2226 to 2440 kg/m^3^. It was found in this study that the density decreased with an increase in the CAA percentages used as a partial replacement for cement. This can be attributed to the fact that CAA has a lower specific gravity compared to OPC. The result is in agreement with Elinwa and Ejeh^31^, who observed that when cement is partially replaced by wood waste ash in mortar mixes, there is a reduction in density, which becomes more significant at higher replacement percentages. According to the study by Chowdhury et al.^34^, the bulk density of grade 20 concrete mix was reduced to 2281 kg/m^3^ at 40% replacement of wood ash. This was attributed to the fact that wood waste ash has a lower specific gravity compared to OPC. The reduced density is likely to influence the strength properties of the sandcrete block specimens with higher CAA content. Considering the three types of sandcrete blocks based on lightweight (300 to1950 kg/m^3^), normal (2200 to 2400 kg/m^3^), and heavyweight (greater than 2500 kg/m^3^) as indicated by Falade et al.^35^, the result of this study provided the sufficient density of sandcrete block specimens that can be used for normal building construction application.

**Table 3:** Average density of sandcrete block specimens.

CAA content (%)	Mass (kg)	Density (kg/m^3^)	Curing age (day)
0	2.932	2255.385	7
5	2.825	2173.077	
10	2.854	2195.385	
15	2.888	2221.538	
20	2.861	2200.769	
25	2.837	2182.308	
0	2.901	2231.538	14
5	2.855	2196.154	
10	2.824	2172.308	
15	2.840	2184.615	
20	2.817	2166.923	
25	2.910	2238.462	
0	2.975	2288.462	21
5	2.859	2199.231	
10	2.875	2211.538	
15	2.929	2253.077	
20	2.782	2140.000	
25	2.958	2275.385	
0	2.949	2268.462	28
5	2.924	2249.231	
10	2.891	2223.846	
15	2.894	2226.154	
20	2.867	2205.385	
25	2.775	2134.615	

### Water absorption

**Figure 5:**
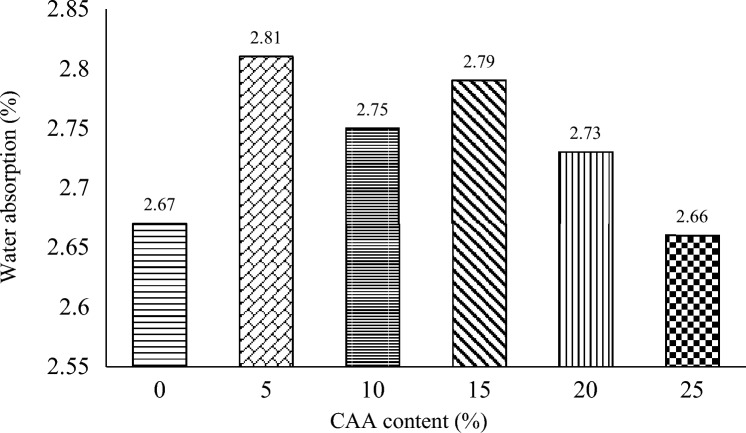
Water absorption results.

Figure 5 presents the results of the water absorption test on the sandcrete block specimens. The results show that the absorption rates vary among the test specimens. It is found that 5% CAA replacement had the highest water absorption of 2.81%, followed by 15% CAA replacement with 2.79%, and the least absorption from 25% CAA replacement with 2.66%. The water absorption rate is slightly lower with 25% CAA replacement (2.66%) as compared with the control (2.67%), implying a better resistance to water uptake, which can be attributed to the fact that more hydration is produced with the higher content of CAA in the sandcrete block specimen. However, it must be noted that the experimental variable for CAA had water absorption (2.73–2.81%) higher than the control. An increase in water absorption occurs when pore spaces in mortars are enlarged; thus, the greater the water absorption, the greater the pore spaces. The water absorption results ranging from 2.66 to 2.81% in this study were better than the water absorption result of 5.25 to 8.25% found in the study by Anowai and Afunanya^12^ on the suitability of millet hush ash (MHA) as partial replacement of cement in the production of sandcrete blocks, 9.89 to12.22% results found in the study by Anowai et al.^7^ on the suitability use of Maize Cob Ash (MCA) as partial replacement of OPC in the production of sandcrete blocks. However, the results of 0.14 to 1.05% obtained by the Udoeyo et al.^36^ study on waste wood ash (WWA) as an additive were lower than the results of this study. The 2.66 to 2.81% water absorption values in this study are far less than the maximum 12% value recommended by BS 2028:2000^25^.

### Compressive strength results

**Figure 6:**
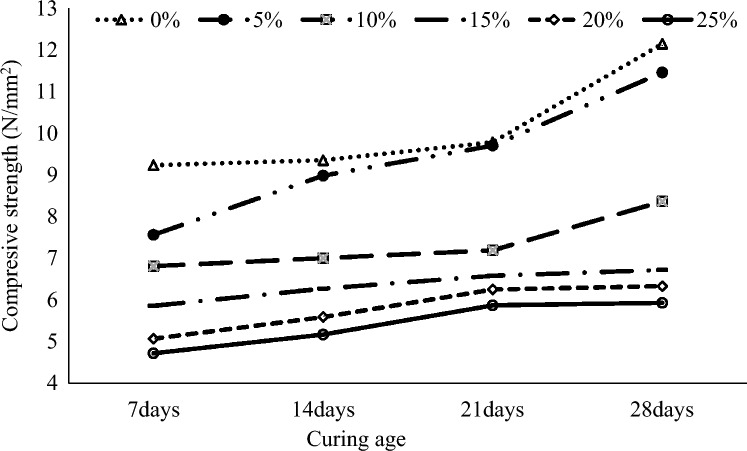
Graphical summary of comprehensive strength of sandcrete block specimens.

The average results of the compressive strength of sandcrete block specimens are summarized in Fig. 6. It can be observed that the compressive strength of the sandcrete block specimens increased with increased curing days for all the specimens. However, the compressive strength of the sandcrete block specimens with CAA replacement of OPC decreased with increased percentage replacement. The decrease can be attributed to the reduced density of the sandcrete block specimens with higher CAA content, and weak bonding between CAA particles and the particle of the other materials in the mortar. At 28 days of curing, the control specimens recorded the highest compressive strength of 12.14 N/mm^2^, followed by 11.45 N/mm^2^ at 5% CAA partial replacement for cement, and the least was 5.93 N/mm^2^ at 25% CAA partial replacement of cement. A study by Abubakar et al.^37^ used cassava peel ash (CPA) to replace a percentage of the cement and recorded a compressive strength of 2.16–2.67 N/mm^2^. Ikeagwuani et al.^38^ studied the optimization of sandcrete blocks with coconut shell ash (CSA) as a partial replacement for cement and achieved the highest compressive strength at a 5% replacement level with 4.07 N/mm^2^. Mahmoud et al.^39^ used groundnut shell ash (GSA) in their study to replace cement and found that the compressive strength of sandcrete bricks with groundnut shell ash (GSA) as a cement replacement ranges from 4.50 to 0.26 N/mm^2^. This indicates that the compressive strengths of the sandcrete block specimens obtained in this study are satisfactory. After 28 days of curing the compressive strengths of sandcrete block specimens with 5% to 25% OPC replacement levels were recorded between 4.72 to 11.45 N/mm^2^, respectively. These values exhibit compressive strengths far above the recommended values of 3.5 and 2.8 N/mm^2^ by the British standard BS EN 772-1:2011^27^, and the Ghana Standard GS 766:2011^20^, respectively.

### Split tensile strength results

**Figure 7:**
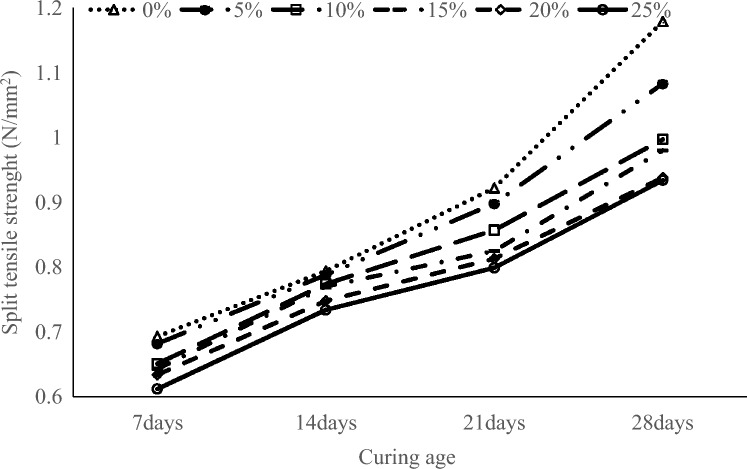
Graphical summary of tensile strength of sandcrete block specimens.

The results of the sandcrete block specimens are graphical summarised is shown in Fig. 7. It can be observed that at 28 days of curing, the control specimens recorded the highest tensile strength of 1.179 N/mm^2^, followed by 1.083 N/mm^2^ obtained by 5% CAA partial replacement of cement, and the least was 0.934 N/mm^2^ at 25% CAA partial replacement of cement. This trend is consistent with the compressive strength results. These results are similar to those of Akeke et al.^40^ who recorded 0.91 to 1.94 N/mm^2^ on the effects of replacing ordinary Portland cement with rice husk ash (RHA) after 28 days of curing. The tensile strength of this study was lower than the average tensile strength of 2.52 to 4.06 N/mm^2^ reported by Mayooran et al.^41^ on open-air burning of low and high-carbon rice husks to manufacture cement blocks. The results show that the split tensile strength of sandcrete block specimens increased with increased curing age, but the tensile strength of the specimens declined with increasing CAA replacement of cement. This reduction can be attributed to the reduced density of the sandcrete block specimens with higher CAA content, and weak bonding between CAA particles and the particle of the other materials in the mortar. The result of the study satisfied the minimum 0.05 N/mm^2^ tensile splitting strength of the specimen recommended by BS EN 12390-6:2000^42^.

## Conclusions

This study aimed at investigating the properties of sandcrete blocks produced with cashew apple ash (CAA) as a replacement for cement. The findings of the study are summarized as follows:

i. The sum of Silicon Oxide (SiO_2_), Aluminium Oxide (Al_2_O_3_), and Iron Oxide (Fe_2_O_3_) obtained in CAA was found to be to be at the level of a pozzolanic material for cement replacement.
ii. The average density of sandcrete block stabilized with CAA as a partial replacement for cement obtained in this study was found to be sufficient for sandcrete blocks that can be used for normal building construction applications.
iii. Water absorption rates of the sandcrete block specimens for this study was found to be far better than recommended value.
iv. The compressive strengths of the sandcrete block specimens obtained in this study were far above the recommended values.
v. The results of the split tensile strength of the specimen was found to be better with a 5% CAA replacement level.

The study concludes that CAA can be used as a replacement for cement in sandcrete block production for normal construction applications. It is recommended that block manufacturers use 5% CAA replacement of cement in manufacturing sandcrete blocks for construction application.

## Data Availability

The data that support the findings of this study are available on request from the corresponding author.

## References

[1] Mansur SA, Hamid AA, Yusof NA (2016). Rising trend in construction cost and housing price. J. Adv. Res. Bus. Manag. Stud..

[2] Jegele TO, Adanikin A (2018). Assessment of the strength of conventionally produced sandcrete blocks and burnt bricks. IOSR J. Mech. Civil Eng..

[3] Danso H, Obeng-Ahenkora NK (2018). Major determinants of prices increase of building materials on ghanaian construction market. Open J. Civil Eng..

[4] Ettu LO, Nwachukwu KC, Arimanwa JI, Anyanwu TU, Okpara SO (2013). Strength of blended cement sandcrete & soilcrete blocks containing afikpo rice husk ash and corn cob ash. Int. J. Modern Eng. Res..

[5] Anosike MN, Oyebade AA (2012). Sandcrete blocks and quality management in Nigeria Building Industry. J. Eng. Project Prod. Manag..

[6] Olubajo OO, Odey OA, Abdullahi B (2019). Potential of orange peel ash as a cement replacement material. Traektoriâ Nauki Path Sci..

[7] Anowai SI, Ishaya AA, Goh DJ, Yahaya AM, Oyelade OM (2020). Suitability of maize cob ash as a partial replacement of cement in sandcrete blocks. Int. J. Environ. Stud. Saf. Res..

[8] Manasseh J (2010). A review of partial replacement of cement with some agro wastes. Niger. J. Technol..

[9] Apata AO, Alhassan AY (2012). Evaluating locally available materials as partial replacement for cement. J. Emerg. Trends Eng. Appl. Sci..

[10] Olawale SO, Tijani MA (2019). Compressive strength of cocoa pod ash blended sandcrete blocks produced in Osogbo, Nigeria. Ann. Fac. Eng. Hunedoara.

[11] Arasa FO, Onchiri RO (2017). Effect of sugarcane Bagasse ash on the engineering properties of blended sandcrete blocks. Int. J. Eng. Res. Technol..

[12] Anowai SI, Afunanya JE (2017). Millet husk ash as partial replacement of cement in sandcrete block. Int. Res. J. Eng. Technol..

[13] Akyereko YG, Wireko-Manu FD, Alemawor F, Adzanyo M (2022). Cashew apples in Ghana: Stakeholders’ knowledge, perception, and utilization. Int. J. Food Sci..

[14] Wongnaa CA, Awunyo-Vitor D (2013). Profitability analysis of cashew production in Wenchi municipality in Ghana. Botswana J. Agric. Appl. Sci..

[15] Statista. Volume of shelled and unshelled cashew nuts exported from Ghana from 2010 to 2021. https://www.statista.com/statistics/1297303/volume-of-cashew-nut-exports-from-ghana/ (2023).

[16] Preethi, P., Dagadkhair, R.A., Shobana, A., Vanitha, K. Cashew apple processing. Training manual on Cashew Production and Post Harvest Technologies. ICAR-Directorate of Cashew Research, Puttur, Karnataka, India. 143p, 123. (2020).

[17] Oyebisi S, Owamah H, Alomayri T, Ede A (2022). Modelling the strength of cashew nutshell ash-cement-based concrete. Magazine Concrete Res..

[18] Oyebisi S, Ede A, Owamah H, Igba T, Mark O, Odetoyan A (2021). Optimising the workability and strength of concrete modified with anacardium occidentale nutshell ash. Fibers.

[19] Oyebisi S, Igba T, Oniyide D (2019). Performance evaluation of cashew nutshell ash as a binder in concrete production. Case Stud. Constr. Mater..

[20] British Standard (BS) 12:1996—Specifications for Portland Cement. British Standards Institution, London

[21] Ghana Standard (GS) 766:2011—Building and Construction Materials-Specification for Ordinary Portland Cement CEM I

[22] British Standard (BS) 3148: Part 2: 1980—Test of Water for Making Concrete, British Standards Institution, London.

[23] Haustein, E., Kuryłowicz-Cudowska, A. Effect of Particle Size of Fly Ash Microspheres (FAMs) on the Selected Properties of Concrete. *Minerals*, 12, 847. 10.3390/min12070847 (2022)

[24] American Society of Testing and Materials (ASTM) C618-19—Standard specification for coal fly ash and raw or calcined natural pozzolan for use in concrete. p. 1-5. (2019).

[25] British Standards (BS) 2028—Precast Concrete Blocks. British Standard Institute, London. (2000).

[26] British Standard (BS) 1881-122—Testing concrete. Method for determination of water absorption. British Standards Institution, London (2011).

[27] BS EN 772-1—Methods of test for masonry units. Determination of compressive strength. British Standards Institution, London (2011).

[28] British Standards (BS EN) 12390-6—Testing hardened concrete. Tensile splitting strength of test specimens. British Standards Institution, London (2009).

[29] American Society of Testing and Materials (ASTM) C311-07—Standard Test Methods for Sampling and Testing Fly Ash or Natural Pozzolans for Use in Portland-Cement Concrete.

[30] Afolayan JO, Oriola FOP, Moses G, Sani JE (2017). Investigating the effect of eggshell ash on the properties of sandcrete block. Int. J. Civil Eng. Constr. Estate Manag..

[31] Elinwa AU, Ejeh SP (2004). Effects of the incorporation of sawdust waste incineration fly ash in cement pastes and mortars. J. Asian Archit. Build. Eng..

[32] Raheem AA, Momoh A, Soyingbe AA (2012). Comparative analysis of sandcrete hollow block and laterite interlocking blocks as walling elements. Int. J. Sustain. Constr. Eng. Technol..

[33] Kumari S, Chander D, Walia R (2018). Durability and strength analysis of concrete by partial replacement of cement with corn cob ash and rice. Int. J. Res. Advent Technol..

[34] Chowdhury S, Mishra M, Suganya O (2015). The incorporation of wood waste ash as a partial cement replacement material for making structural grade concrete: An overview. Ain Shams Eng. J..

[35] Falade F, Ikponmwosa E, Arogundade A (2011). Investigation of some structural properties of foamed aerated concrete. J. Eng. Res..

[36] Udoeyo FF, Inyang H, Young DT, Oparadu EE (2006). Potential of wood ash waste as an additive in concrete. J. Mater. Civil Eng..

[37] Abubakar J, Anum B, Tsonde R (2021). Use of Cassava Peel Ash (CPA) in the production of hollow, non-load bearing sandcrete blocks. J. Eng. Sci. Technol..

[38] Ikeagwuani C, Udokpoh U, Onyia ME (2020). Optimisation of Compressive Strength of Sancrete Block Containing Coconut Shell Ash as Cement Partial Replacement.

[39] Mahmoud H, Belel ZA, Nwakaire C (2012). Groundnut shell ash as a partial replacement of cement in sandcrete blocks production. Int. J. Dev. Sustain..

[40] Akeke GA, Ephraim ME, Akobo IZS, Ukpata JO (2013). Structural properties of rice husk ash concrete. Int. J. Eng..

[41] Mayooran S, Ragavan S, Sathiparan N (2017). Comparative study on open air burnt low-and high-carbon rice husk ash as partial cement replacement in cement block production. J. Build. Eng..

[42] British Standard (BS EN) 12390-Testing hardened concrete—Part 2: Making and curing specimens for strength tests. British Standards Institution, London (2000).

